# Factors Influencing Sleep Quality among Female Staff Nurses during the Early COVID-19 Pandemic in the United States

**DOI:** 10.3390/ijerph18094827

**Published:** 2021-04-30

**Authors:** Yeoun Soo Kim-Godwin, Meen Hye Lee, Jeongok G. Logan, Xiaoyue Liu

**Affiliations:** 1 School of Nursing, University of North Carolina Wilmington, 601 South College Rd., Wilmington, NC 28403, USA; kimy@uncw.edu; 2 School of Nursing, University of Virginia, 225 Jeanette Lancaster Way, Charlottesville, VA 22903, USA; jl3zj@virginia.edu (J.G.L.); xl5tb@virginia.edu (X.L.)

**Keywords:** nurse, sleep quality, work environment, COVID-19

## Abstract

This study aimed to assess the overall level of sleep quality among female staff nurses in the United States during the early COVID-19 pandemic. It also aimed to examine factors associated with sleep quality and its seven subcomponents: subjective sleep quality, sleep latency, sleep duration, habitual sleep efficiency, sleep disturbance, use of sleeping medications, and daytime dysfunction. A descriptive, correlational, and cross-sectional study design was used. We performed descriptive, and regression analyses with a sample of 215 female staff nurses enrolled in post-licensure online nursing programs at a southeastern state university. Data collection was conducted using an online survey from April to May 2020. Sleep quality was measured using the Pittsburgh Sleep Quality Index (PSQI). Nurses working part time (*p* = 0.02), with lower perceived physical health (*p* = 0.01), a lower self-care self-regulation score (*p* < 0.001), and higher work stress (*p* < 0.05) showed poorer sleep quality. Factors associated with subcomponents of sleep quality varied. Poor sleep quality among nurses during the COVID-19 pandemic was reported. Various factors, including work environmental factors were associated with the sleep quality in this sample. Hospital administrators should consider developing intervention programs for improving the work environment, which would impact sleep quality, health status, and job performance.

## 1. Introduction

Sleep quality is crucial for nurses to provide optimal care to patients. However, due to the nature of the work, nurses are at a higher risk for decreased sleep quantity and quality, as well as continuous sleep deprivation [1]. Fatigue resulting from poor sleep tends to reduce nurses’ ability to concentrate and make correct decisions, leading to the possibility of errors and injuries [2,3]. For example, risk of medication errors was associated with poor quality of sleep among nurses [4]. In addition, poor sleep quality was related to a decreased quality of life in nurses. Female nurses who reported a good quality of life had significantly higher reported sleep quality than those with moderate or poor quality of life [5].

Previous studies reported that many factors might cause poor sleep for nurses, including physical and psychosocial work strain, as well as personal factors (e.g., family responsibilities, age, gender, race, marital status, and socio-economic status) [1]. Women have reported a higher risk of suffering from insomnia and experiencing difficulty in falling asleep [6,7]. Sleep trouble has also been linked to self-care factors, such as lack of physical activity [8] and poor health outcomes, including obesity or chronic disease and pain [9,10]. Body mass index (BMI) was found to be a significant predictor of sleep patterns, with a one-unit increase (kg/m^2^) leading to a 19% decrease in sleep quality [11].

Work-related factors were found to be associated with sleep problems among nurses. Shift work has been consistently identified as a contributor to poor sleep for nurses [3]. Rotating shift work was reported as a primary cause of sleep disorders, with night shift nurses reporting significantly higher scores of poor overall sleep quality than day shift nurses [11,12]. Moreover, many studies reported that work stress was associated with poor sleep quality. For example, sleep disturbances among Chinese community nurses were highly associated with job stress, doctor–patient relationships, psychosomatic state, environmental factors, and promotion or competition [13].

The current study examined factors associated with sleep quality among female staff nurses in the United States. The perception of sleep quality is complex and related to various subjective factors. While previous studies reported some of these associations, they tended to focus primarily on work stress and sleep quality; however, it is worth analyzing these variables together, which incorporate a wider range of factors. Additionally, the current study aimed to assess the level of overall sleep quality for staff nurses, who were the first line of bedside care providers during the early COVID-19 pandemic [14].

## 2. Methods

### 2.1. Sample and Procedure

This descriptive, correlational study was conducted at a public state university in the southeastern United States. The current study analyzed 215 female staff nurses enrolled in post-licensure online nursing programs at the university. The study received approval from the university Institutional Review Board. Email invitations to participate in the online survey were sent to RNs (registered nurses) enrolled in post-licensure (RN-BSN, MSN, and DNP) programs. Qualtrics, a secure online survey platform, was used to collect survey responses anonymously. The online survey was disseminated over a 3-week period during the COVID-19 pandemic (19 April to 6 May 2020). Three reminder emails were sent out to prompt responses. All emails contained the contact information for the researcher and an explanation that the survey was voluntary.

### 2.2. Measures

The following sociodemographic and biometric variables were included in the online survey: age, race and ethnicity (White, African American, Hispanic, and Other), BMI (calculated by self-reported height and weight), highest degree of education (categorized by diploma, associate, bachelor’s, and master’s degrees), work status (full-time vs. part-time), shift (categorized by day vs. non-day shift groups including evening, night, and rotating shifts), years of work experience as an RN, and perceived physical health (measured by a single question rating health status as poor, fair, good, or excellent).

We modified the Self-Care Self-Regulation Questionnaire (SCSRQ) for this study [15]. This questionnaire was selected because it reflects the duties of nurses by the American Nurses Association (ANA) Code of Ethics [16]. While the original SCSRQ has 32 items, the modified SCSRQ consists of 20 items to evaluate self-care in the four same domains, including personal growth (6 items), healthy lifestyle (6 items), control over negative mood (4 items), and control over health risks (4 items). Respondents rated each item on a 5-point Likert scale ranging from 1 (not typical for me) to 5 (very much typical for me) [15]. The modified SCSRQ as a whole and its individual subscales demonstrated satisfactory psychometric properties. The Cronbach’s alpha of the current 20-item questionnaire was 0.91, while the original 34-items was 0.89 [15].

Work stress was assessed using 8 items selected from the Nursing Stress Scale (NSS) [17]. The original NSS consists of 34 items with 7 subscales that describe situations identified as causing stress for nurses in the performance of their duties [17]. From the NSS, the current study included 8 items that are specifically related to work: (1) shift hours, (2) conflict with other nurses, (3) conflict with physicians, (4) conflict with other health professionals, (5) lack of support, (6) inadequate preparation of care, (7) meeting the needs, and (8) workplace bullies. Like the NSS, the current study used a 4-point Likert scale to rate how often respondents found such situations stressful (0 = never to 3 = very frequently). The Cronbach’s alpha of the current 8-item questionnaire was 0.81, while the original 34-items was 0.89 [17].

The Pittsburgh Sleep Quality Index (PSQI) was used to assess overall sleep quality. PSQI is a widely used self-reported measure of sleep [18]. The PSQI is a 19-item, self-reported measure of subjective sleep over the past month [19]. Items are rated on a 4-point Likert scale from 0 (not during the past month) to 3 (three or more times a week). It consists of seven components of duration, habitual sleep efficiency, sleep onset latency, sleep disturbance, use of sleep medication, and daytime dysfunction. The seven component scores are added to yield one global PSQI score, which ranges from 0 to 21 [19]. Cutoff scores were used to classify “Good SQ” (sleep quality) (PSQI < 5), and “Poor SQ” (PSQI ≥ 5) [19]. The overall scale reported a Cronbach’s alpha of 0.66 in this study.

### 2.3. Data Analysis

Descriptive statistics were calculated using percentages and means. Multivariate regression analyses were used to identify factors associated with sleep quality using a total score and subcomponents of PSQI. Some of the variables were dichotomized in the regression models: race (white vs. non-white), work shift (day shift vs. else including evening, night, and rotating shifts), and education (diploma/associate degrees vs. bachelor’s/master’s degrees). Multiple imputations were performed for the missing values of total PSQI and each subcomponent. A *p*-value less than 0.05 (two-sided) was defined as statistically significant. The Statistical Package for the Social Science (SPSS) version 26 software (IBM SPSS Inc., Chicago, IL, USA) was used. were used. 

## 3. Results

### 3.1. Demographics

Among the 215 female staff nurses, the majority were whites (83.79%) and worked full time (82.7%). The mean age of participants was 36.1 years old, while the mean RN experience was 6.1 years. The highest degree of education for the majority of the participants was an associate degree (60.9%), and the most common shift was the day shift (57.2%). The average BMI was 29. About 62% of the participants rated their health as good; however, only 13.5% reported their health as ‘excellent’ (Table 1).

### 3.2. Self-Care Self-Regulation

Overall, respondents generally tried to practice self-care (M = 4.04, SD = 0.48) on a five-point Likert scale. The subscale of ‘personal growth’ was the most highly rated (M = 4.32, SD = 0.47). In addition, respondents also rated highly the subscale of ‘control over negative mood’ (M = 4.08, SD = 0.57), showing a high level of self-care for psychological well-being domain items. While the ‘control over health risks’ subscale received a relatively high rating (M = 4.02, SD = 0.60), the ‘physical care’ subscale was rated lower (M = 3.78, SD = 0.75) (Table 1).

### 3.3. Work Stress

Using a four-point Likert scale (0 = never, 1 = occasionally, 2 = frequently, and 3 = very frequently), respondents rated their work stress for the 8 items from the NSS: ‘shift hours’ (M = 1.85, SD = 1.10) was rated highest, followed by ‘meeting the needs of patients/families’ (M = 1.82, SD = 1.04), ‘lack of support’ (M = 1.73, SD = 1.05), ‘inadequate preparation of care’ (M = 1.43, SD = 0.94), ‘conflict with physicians’ (M = 1.06, SD = 0.83), ‘conflict with other nurses’ (M = 1.02, SD = 0.85), ‘conflict with other health professionals’ (M = 1.0, SD = 0.82), and ‘workplace bullies’ (M = 0.95, SD = 0.99). These findings suggest that shift hour is a major factor, which needs to be resolved (Table 1).

### 3.4. Descriptive Statistics of Pittsburgh Sleep Quality Index (PSQI)

Table 2 describes descriptive statistics of sleep quality and the subcomponents of PSQI. Overall, the average of total sleep quality was reported as 9.85 (SD = 2.72). Habitual sleep efficiency was the highest (M = 2.52, SD = 1.32), followed by daytime dysfunction (M = 1.69, SD = 1.73) and sleep latency (M = 1.37, SD = 0.74).

### 3.5. Multivariate Regression for Variables Associated with PSQI

Results of multivariate regression using total score and subcomponents of PSQI as the dependent variables and 10 factors are presented in Table 3 and Table 4. In this model, nurses working full time (t = 2.41, *p* = 0.02), lower perceived physical health (t = −2.59, *p* = 0.01), a lower self-care self-regulation score (t = −3.58, *p* < 0.001), and higher work stress (t = 2.36, *p* < 0.05) showed poor overall sleep quality (Table 3).

Table 4 represents seven multivariate regression models for each PSQI subcomponent. Significant factors varied across the components. The non-white group was more likely to report sleep duration issues compared to the White group (t = 2.34, *p* < 0.05). Nurses working part-time had higher sleep latency problems (t = 2.047, *p* < 0.05). Shift was the most common significant factor affecting four of the components: subjective sleep quality (t = 2.084, *p* < 0.05), sleep latency (t = 1.97, *p* < 0.05), habitual sleep efficiency (t = −4.25, *p* < 0.001), and daytime dysfunction (t = 4.44, *p* < 0.001). The non-day shift group had problems with subjective sleep quality (t = 2.08, *p* < 0.05), sleep latency (t = 1.99, *p* < 0.05), and daytime dysfunction (t = 4.44, *p* < 0.001), while the day shift group had habitual sleep efficiency problems (t = −4.25, *p* < 0.001). Lower perceived physical health was associated with higher daytime dysfunction (t = −2.66, *p* < 0.01), and lower self-care self-regulation was associated with subjective sleep quality (t = −4.96, *p* < 0.001) and daytime dysfunction problems (t = −4.74, *p* < 0.001). Work stress was associated with sleep latency (t = 2.15, *p* < 0.05), sleep disturbance (t = 2.78, *p* < 0.01), and daytime dysfunction (t = 3.33, *p* < 0.001).

## 4. Discussion

The present cross-sectional study provides descriptive information about sleep quality, as well as significant factors that contributed to poor sleep quality among female nurses during the early stage of the COVID-19 pandemic. The female staff nurses of this study sample demonstrated remarkably poor sleep quality in general. Furthermore, various risk factors influencing poor sleep quality include race (non-white), part-time work, non-day shift work, longer RN experience, poor perceived physical health, a low level of self-care self-regulation, and high work stress. The study findings showed that the mean global PSQI score of this study sample (M = 9.85) was noticeably higher than the ones previously reported. For example, data on nurses (*n* = 103) from two Midwestern hospitals in the United States showed that the mean global PSQI was 5.56 among the day shift nurses and 7.08 among the night shift nurses [11]. Direct comparisons for the levels of sleep quality found in this study with the levels reported in previous studies were not possible. However, considering that the generally accepted criterion for poor sleep quality is more than five, the poor level of sleep quality in this study sample is noteworthy. It is not clear if poor sleep quality is typically observed in the nurse population or whether it is attributable to the COVID-19 pandemic. Studies evaluating sleep duration and quality in nurses are scarce, with studies conducted during the COVID-19 pandemic being especially limited. Available studies in China reported large variations in the average levels of sleep quality. For example, a survey from a total of 123 medical workers reported the average PSQI score of 7.22 during the outbreak of COVID-19 in Wuhan, China [20]. Another Chinese study included 120 frontline medical staff, with 60 working at the COVID-19 designated hospital (experimental group) and 60 working at a non-designated hospital (control group). The total average PSQI of the experimental group in this study was 16.07, indicating an exceptionally poor sleep quality, and even the control group showed a poor sleep quality of 10.49; such findings suggest the possible detrimental impacts of COVID-19 on sleep quality [21].

The current study findings showed that poorly perceived physical health and higher work stress were significant contributors to the high global score of sleep quality. In subcomponent analyses, poorly perceived physical health was associated with daytime dysfunction only, but work stress was a significant predictor of longer sleep latency, sleep disturbances, and daytime dysfunction. While many studies have investigated the effects of sleep on physical and psychological health, the current study showed poorly perceived physical health and work stress contributed to poor sleep, suggesting the bidirectional associations between sleep and health. The study findings were also consistent with the previous findings that increased levels of job stress are associated with poor sleep quality [13]. In addition, physical conditions causing pain, heartburn, frequent urination, or coughing may require medications that can affect sleep, which may also be a significant contributor to poor sleep health [22].

On the other hand, previous studies show that inadequate sleep health causes an imbalance of various hormones to regulate hunger sense, increased blood pressure and insulin resistance, increased risk of obesity, hypertension, and type 2 diabetes [9,10]. Considering that sleep plays a critical role in maintaining physical and psychological health, this study highlights the need for future interventions that focus on alleviating work stress and managing physical conditions that interfere with sleep. Our study showed that a low level of self-care self-regulation was a significant predictor of poor global sleep quality, as well as the subcomponents of subjective sleep quality and daytime dysfunction. These findings were similar to the previous report that motivation for physical exercise and eating behaviors (such as uncontrolled eating and emotional eating) were linked to poor sleep quality in nurses [2]. Self-care can be conceptualized as a self-regulation process that activates health promotion behaviors and inhibits health risk behaviors. Self-care is an important concept not only for physically ill people, but also for the whole population [15]. Nurses can use self-care strategies such as self-regulation to lessen their vulnerability to caregiving fatigue and to improve their well-being and resilience [14]. Currently, many nursing schools acknowledge the importance of self-care and attempt to incorporate knowledge and skills for self-care into the nursing curriculum to prevent burnout syndrome in students [23]. Similar efforts are warranted for nurses, especially senior nurses who may not have been exposed to such curriculums. Sleep is one of the foremost behavioral factors for overall health. The endeavor to promote self-care and to educate the importance of good sleep will encourage nurses to adopt behavioral changes to facilitate sleep health which will, in turn, enhance their psychological and physical health.

Further subcomponent analyses of the PSQI scale showed that several factors associated with each component of seep quality. Although racial differences were not observed in global sleep quality in the current study, being non-white was associated with shorter sleep duration (less than 7 h). Previous studies commonly observed racial disparities in sleep quality and duration, showing that non-white populations are more likely to have poor sleep quality and short sleep duration. A meta-analysis of 14 studies reported that urban residence, unconventional work schedules, and job stress may explain sleep disparities in different ethnicities [22]. Given the increasing evidence on racial disparity in sleep duration consistently observed in this study along with the previous studies, further explorations for factors contributing to short sleep duration in non-white population are recommended.

In our study, neither non-day shift nor work stress was associated with sleep duration. However, non-day shift work had significant associations with poor subjective sleep quality, longer sleep latency, lower habitual sleep efficiency, sleep disturbances, and daytime dysfunction. This finding is consistent with the abundant evidence that those working nights had a poorer score in terms of sleep latency having more time, and difficulty falling asleep [11]. Night shift work disrupts circadian rhythm, causing sleep disorders [11,12]. Sleep disorders also increase the risk of ulcers, insulin resistance, and cardiovascular disease. Nevertheless, working non-traditional hours is inevitable in certain occupations, including nursing [3]. Our study showed an interesting finding that part-time workers have significantly poorer sleep quality and longer sleep latency after controlling for other potential contributors. It is unknown whether or not these part-time workers have multiple jobs. Information for contextual factors of part-time workers would be required to explain this finding. It is also surprising that longer RN experience was associated with sleep disturbances. This might suggest the positive correlation between age and RN experience. Age is a well-known factor contributing to sleep disorder [5]. A number of factors were identified to explain the relationship between age and sleep disorder, including hormonal change, daytime napping, polypharmacy, and illnesses [5]. However, our finding was from the model that adjusted for potential risk factors of sleep disturbance, including age; therefore, explorations for further contextual factors are needed to explain the relationship between longer RN experience and sleep disturbance.

### Limitations

The current study has several limitations due to the use of a convenience sample and the cross-sectional design, thereby only able to identify correlational associations. In addition, the sample consisted of RNs enrolled in post-licensure programs. Nurses who chose to be enrolled in post-licensure programs may have more time constraints and poor sleep quality. Finally, multiple imputations were performed with total PSQI and each of the subcomponents because 168 out of 215 (78.1%) had one or more missing responses for the PSQI questions, which might have caused the lower PSQI reliability level of 0.66 in this study. However, we conducted a multiple imputation procedure for missing data replacement to address the missing points.

## 5. Conclusions

The findings of the current study provide a snapshot of the remarkably poor sleep quality among nurses. Considering that sleep quality in healthcare providers is associated with quality of care, frequency of medical errors, and overall patient safety, such findings warrant urgent interventions for this issue. Educational interventions to emphasize the importance of sleep health and to cultivate tips for sleep hygiene should be incorporated.

Sleep quality was influenced by nurses’ part-time status, non-day shift, poor perceived physical health, a low level of self-care self-regulation, and a high level of work stress. Considering that sleep plays a crucial role in maintaining physical and psychological health, this study highlights the need for future interventions that focus on alleviating work stress and managing physical conditions that interfere with sleep. Therefore, it is essential to propose intervention programs for improving the work environment while encouraging self-care. At the individual level, nurses need to make an effort to implement self-care practices. Moreover, institutions need to offer programs for nurses to develop desirable sleep strategies, self-care, and stress management at work. Institutions also need to develop programs for non-day nurses, considering this type of work increases diverse sleep issues. Hospital administrators should advocate for a flexible policy to accommodate non-day shift workers’ needs.

## Figures and Tables

**Table 1:** Demographic and Health Characteristics of Female Staff Nurses (*n* = 215).

Variable	Frequency	Mean	Range	SD
**Age (year)**		36.07	19–65	10.24
**Highest Level of Education**				
Diploma	7 (3.3%)			
Associate degree	131 (60.9%)			
Bachelor’s Degree	74 (34.4%)			
Master’s Degree	3 (1.4%)			
**Ethnicity**				
White	180 (83.7%)			
African American	19 (8.8%)			
Asian	4 (1.9%)			
Hispanic	10 (4.7%)			
Other	2 (0.9%)			
**Work**				
Full time	177 (82.7%)			
Part-time	37 (17.2%)			
**Shift**				
Day shift	123 (57.2%)			
Evening shift	12 (5.6%)			
Night shift	62 (28.8%)			
Rotating shift	18 (8.4%)			
**Number of years as an RN (years)**		6.87	0.5–41	7.02
**Self-rated health**				
Poor	3 (1.4%)			
Fair	49 (22.8%)			
Good	134 (62.3%)			
Excellent	29 (13.5%)			
**BMI**		29.11	18.5–68.4	7.22
**Self-care self-regulation (total score)**		4.04	2.81–5	0.48
Personal growth		4.32	2.84–5	0.47
Control over negative mood		4.08	2.25–5	0.57
Physical self-care		3.78	1.57–5	0.75
Control over health risks		4.02	2.25–5	0.60
**Work Stress (total score)**		1.36	0–2.88	0.63
Shift hours		1.85	0–4	1.10
Conflict with other nurses		1.02	0–4	0.85
Conflict with physicians		1.06	0–4	0.83
Conflict with other health professions		1.01	0–4	0.82
Lack of support		1.73	0–4	1.05
Inadequate preparation of care		1.43	0–4	0.94
Meeting the needs of patients/families		1.82	0–4	1.04
Workplace bullies		0.95	0–4	0.99

Note. Because of missing data, the total was not 215 for each item.

**Table 2:** Descriptive Statistics of Pittsburgh Sleep Quality Index Scores (*n* = 215).

	Mean	Standard Deviation
**PSQI (Total Score)**	**9.85**	**2.72**
**Sub-components**		
Subjective sleep quality (Comp 1)	1.37	0.74
Sleep latency (Comp 2)	1.62	1.23
Sleep duration (Comp 3)	1.09	0.84
Habitual sleep efficiency (Comp 4)	2.52	1.32
Sleep disturbances (Comp 5)	1.40	0.67
Use of sleeping medications (Comp 6)	0.78	1.35
Daytime dysfunction (Comp 7)	1.69	1.73

Note. Because of missing data, PSQI score and the subcomponents were obtained from multiple imputation data.

**Table 3:** Multivariate regression for variables associated with PSQI (*n* = 215).

Variable	β	SE	t	*p*
Age	0.008	0.030	0.257	0.797
Education ^†^				
Diploma/Associate	0.048	0.472	0.103	0.918
Race ^††^				
Non-White	0.222	0.591	0.375	0.708
Work ^†††^				
Part-time	0.146	0.605	2.408	**0.016**
Shift ^††††^				
Non-day shift	0.604	0.444	1.360	0.174
RN experience	0.056	0.040	1.403	0.161
Perceived physical health	−1.064	0.411	−2.591	**0.010**
BMI	0.011	0.034	0.305	0.760
Self-care self-regulation	−0.094	0.026	−3.577	**0.000**
Work Stress	0.893	0.378	2.364	**0.018**

Note*:* ^†^ Reference: Bachelor/Master; ^††^ Reference: White; ^†††^ Reference: Full-time; ^††††^ Reference: Day shift. Bold indicates statistically significant. SE = Standard Error.

**Table 4:** Multivariate regression for variables associated with sub-components of PSQI (*n* = 215).

Variable	Subjective Sleep Quality		Sleep Latency		Sleep Duration		Habitual Sleep Efficiency		Sleep Disturbances		Use of Sleeping Medications		Daytime Dysfunction	
	β	SE	β	SE	β	SE	β	SE	β	SE	β	SE	β	SE
Age	−0.001	0.006	−0.001	0.009	0.008	0.009	0.004	0.011	−0.003	0.005	0.002	0.011	−0.002	0.006
Education ^†^														
Diploma/Associate	0.016	0.103	−0.041	0.149	0.022	0.142	0.079	0.168	0.053	0.084	0.035	0.171	−0.116	0.103
Ace ^††^														
Non-White	0.051	0.130	0.211	0.186	**0.384** *	0.164	−0.081	0.212	−0.009	0.103	−0.208	0.214	−0.125	0.129
Work ^†††^														
Part-time	0.153	0.133	**0.390** *	0.191	0.267	0.170	−0.026	0.213	0.160	0.110	0.371	0.219	0.142	0.133
Shift ^††††^														
Non-day shift	**0.202** *	0.097	**0.278** *	0.140	0.101	0.142	**−0.686** ***	0.161	**0.004** *	0. 075	0.274	0.160	**0.431** ***	0.097
RN experience	0.014	0.009	0.016	0.012	0.011	0.012	−0.013	0.014	**0.015** **	0.007	0.013	0.014	0.000	0.009
Perceived physical health	−0.019	0.088	0.125	0.127	−0.199	0.133	−0.036	0.146	−0.190	0.071	−0.261	0.146	**−0.234** **	0.088
BMI	0.006	0.007	0.012	0.011	−0.040	0.011	−0.005	0.012	0.006	0.006	−0.006	0.012	−0.002	0.007
Self-care self-regulation	**−0.028** *	0.006	−0.015	0.008	−0.012	0.008	0.008	0.010	−0.007	0.005	−0.014	0.010	**−0.027** ***	0.006
Work Stress	0.119	0.081	**0.248** *	0.116	0.145	0.102	−0.037	0.155	**0.177** **	0.064	−0.027	0.133	**0.269** **	0.081

Note: *p* values, * < 0.05, ** < 0.01, *** < 0.001, ^†^ Reference: Bachelor/Master; ^††^ Reference: White; ^†††^ Reference: Full-time; ^††††^ Reference: Day-shift, SE = Standard Error, Bold indicates statistically significant.

## Data Availability

The data presented in this study are available on request from the corresponding author.
